# eIF3a‐PPP2R5A‐mediated ATM/ATR dephosphorylation is essential for irinotecan‐induced DNA damage response

**DOI:** 10.1111/cpr.13208

**Published:** 2022-02-21

**Authors:** Chao Mei, Ze‐En Sun, Li‐Ming Tan, Jian‐Ping Gong, Xi Li, Zhao‐Qian Liu

**Affiliations:** ^1^ Department of Clinical Pharmacology Hunan Key Laboratory of Pharmacogenetics National Clinical Research Center for Geriatric Disorders Xiangya Hospital Central South University Changsha China; ^2^ Institute of Clinical Pharmacology Engineering Research Center for Applied Technology of Pharmacogenomics of Ministry of Education Central South University Changsha China; ^3^ Department of Pharmacy The Second People’s Hospital of Huaihua City Huaihua China

**Keywords:** chemoresistance, DNA damage response, eIF3a, irinotecan, PPP2R5A

## Abstract

**Objectives:**

The individual differences and pervasive resistance seriously hinder the optimization of irinotecan‐based therapeutic effectiveness. Eukaryotic translation initiation factor 3a (eIF3a) plays a key role in tumour occurrence, prognosis and therapeutic response. This study focused on the role of eIF3a in irinotecan‐induced DNA damage response.

**Materials and Methods:**

The cck8 cell viability and clone survival analyses were used to test the regulatory role of eIF3a on irinotecan sensitivity in HT29 and CACO2 cell lines in vitro. This regulatory role was also verified in vivo by conducting subcutaneous xenograft model. Irinotecan‐induced DNA damage, cell cycle arrest and apoptosis were tested by flow cytometry analysis, TUNEL staining, western blot and comet assays. The immunofluorescence, co‐IP, luciferase reporter assay, RIP and flow cytometric analyses were carried out to investigate the underline mechanism.

**Results:**

We demonstrated that eIF3a continuously activates ATM/ATR signal by translationally inhibiting PPP2R5A, a phosphatase that directly dephosphorylates and inactivates ATM/ATR after DNA repair complete. Suppression of PPP2R5A resulted in chronic ATM/ATR phosphorylation and activation, impairing DNA repair and enhancing irinotecan sensitivity.

**Conclusions:**

Our study suggested eIF3a with a high potential to influence phenotypic functions, which may contribute substantially to the early identification of susceptible individuals and the provision of personalized medication to irinotecan‐treated patients.

## Graphical abstract

Schematic diagram for eIF3a regulates the sensitivity of irinotecan. Left, camptothecin and its derivatives, including irinotecan, rapidly induce SSBs in malignant cells. The SSBs would convert into DSBs when encountering the replicating forks. The existence of DNA lesions rapidly trigged DNA damage response, which was initiated by autophosphorylation of ATM and ATR. The activation of ATM/ATR signal sequentially activates the downstream effectors to control a series of DNA damage response like cell cycler arrest, DNA repair and apoptosis signal. Right, once the repair process finished, specific protein phosphatases including PPP2R5A are involved in the dephosphorylation of p‐ATM and p‐ATR so as to promote checkpoint recovery. eIF3a participates in this process by translationally regulating PPP2R5A. Suppression of PPP2R5A induces persistent ATM and ATR phosphorylation and activation, leading to impaired DNA damage repair and cell death.

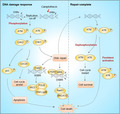

## 1 INTRODUCTION

Colorectal cancer is the third prevalent cancer and the second leading cause of tumor‐related mortality worldwide.^1^ Current methods for colorectal cancer therapy rely heavily on DNA‐damaging agents. Irinotecan, a semisynthetic derivative of camptothecin, has been approved for the first line treatment of metastatic colorectal cancer.^2^ Irinotecan must first be metabolized by carboxylesterase (CES) to generate an SN38 (yield 7‐ethyl‐10‐hydroxy camptothecin),^3^ which is an active metabolite that specifically inhibits topoisomerase I (Top1) by forming Top1–DNA covalent complexes (Top1cc) to block the DNA religation step and generate DNA single strand breaks (SSBs). The SSBs would convert into DNA double strand breaks (DSBs) when they encounter replication forks and finally lead to cell apoptosis.^4^ Irinotecan‐induced DNA damage rapidly triggers DNA damage response, an indispensable mechanism activated by ataxia‐telangiectasia‐mutated (ATM) and ataxia‐telangiectasia and RAD3‐related (ATR)‐dependent phosphorylation of several downstream targets such as H2AX and Chk1/Chk2.^5^ Cell genome stability is maintained by DNA damage response by integrally coordinating DNA repair activity, cell cycle checkpoint, γ‐H2AX signalling and apoptosis program,^6^, ^7^, ^8^ all of which may greatly affect the therapeutic effect and tumour response to irinotecan.^9^, ^10^

Eukaryotic translation initiation factor 3 (eIF3) is the most complex eukaryotic translation initiation factor that consisted of 13 subunits (eIF3a to eIF3m). eIF3a is a highly conserved 170‐kDa protein that needed in mRNA translation initiation. It widely participates in DNA synthesis and repair, cell growth, cell cycle, fibrosis, drug resistance and several other signalling pathways.^11^ Furthermore, eIF3a is overexpressed in several types of cancers,^12^, ^13^, ^14^, ^15^, ^16^, ^17^, ^18^, ^19^, ^20^ indicating a special role in carcinogenesis. Knocking down of eIF3a has been reported to enhance two classical DNA‐damaging agents, platinum‐ and anthracycline‐ based chemotherapy resistance, by regulating the nucleotide excision repair (NER) and non‐homologous end joining (NHEJ) repair, respectively.^21^, ^22^ These findings indicate that eIF3a may play a vital role in tumour progression as well as the therapeutic response of tumour patients.

Presently, widespread resistance extensively limits the use of chemotherapy medications. To acquire better outcomes, novel and credible biomarkers are urgently needed for future research. This study demonstrated for the first time that eIF3a negatively regulates irinotecan sensitivity in colorectal cancer. Specifically, eIF3a translationally regulates protein phosphatase 2, regulatory subunit B (B56), alpha isoform (PPP2R5A), a phosphatase that we proved to directly dephosphorylate p‐ATM and p‐ATR. Suppression of PPP2R5A leads to prolonged DNA damage response signal and impaired repair process. This study provides a potential therapeutic target for early identification of different susceptible patients, allowing for provision of personalized medication to suitable individuals.

## 2 MATERIALS AND METHODS

### 2.1 Cell culture and transfection

Two human colorectal cancer cell lines Caco2 and HT29, as well as human embryonic kidney 293T cells, were obtained from the cell banks of the Shanghai Institutes of Biological Sciences and maintained at 37℃ in a 5% CO_2_‐humidified incubator. McCoy's 5A medium, RPMI‐1640 medium and DMEM were used to culture HT29, Caco2 and 293T cells, respectively. The medium was supplemented with 10% foetal bovine serum (FBS; Biotechnology). The medium was supplemented with 10% FBS (BI). The specific small interfering RNAs (siRNAs) for silencing eIF3a or PPP2R5A were synthesized by Ribobio, and the sequences are provided in Table S1. The cellular transfection was performed using Lipofectamine RNAiMAX (Invitrogen) reaction system according to the protocol. The eIF3a plasmid was transfected using Lipofectamine 3000 Reagent (Invitrogen) to overexpress eIF3a following the manufacturer’s instructions.

### 2.2 RNA isolation and RT‐PCR

Total RNA was isolated with Trizol reagent (Takara) following the manufacturer's instruction. The reverse transcription of RNA into cDNA was conducted using PrimeScript^TM^ RT reagent kit (Takara) according to the protocol. Quantitative reverse transcriptase polymerase chain reaction (RT‐PCR) assay was performed on LightCycler^®^ 480 PCR system (Roche). The relative mRNA expression was calculated by the $2^{‐\Delta \Delta C_{T}}$ method. The primer sequences used in this study are listed in Table S2.

### 2.3 Western blot analysis

The whole‐cell lysates were obtained with RIPA lysis buffer. Protein concentration was determined using the bicinchoninic acid method based on the manufacture's protocol. Cell protein lysates were first separated using SDS‐PAGE before being transferred to PVDF membranes (Millipore). The membranes were incubated in 5% skim milk for 2 h at room temperature before being incubated in specific primary antibodies at 4°C overnight. Next, the membranes were incubated with secondary antibodies for 1 h at room temperature. Protein bands were detected using ECL method. The eIF3a, caspase‐3, cleaved caspase‐3 (Asp175), parp, cleaved parp (Asp214), caspase‐9, cleaved caspase‐9 (Asp330), caspase‐7, cleaved caspase‐7 (Asp198), anti‐rabbit IgG, γ‐h2AX and ATR antibodies were obtained from CST. The ATM, phospho‐ATM (S1981), PPP2R5A and Ki67 antibodies were procured from Abcam. The phospho‐Cdc25c (S216), phospho‐Chk1 (S317) and phospho‐ Chk2 (T68) antibodies were purchased from Proteintech. The Chk1 and Chk2 antibodies were supplied by Santa Cruz Biotechnology. The phospho‐ATR (T1989) antibodies were purchased from Abclonal Technology.

### 2.4 Cell viability analysis

The HT29 and Caco2 cells were transfected in six‐well plates before being seeded overnight in 96‐well plates (Corning) at a density of 3  × 10^3^ cells per well. Irinotecan (Sigma) was dissolved in dimethyl sulfoxide in a concentration of 100 mM and stored at −80°C. The cells were incubated with gradient concentration of irinotecan for 48 h. Cell viability was tested using CCK8 method (Bimake) in accordance with the manufacturer's protocol. For each well, 10 µl CCK8 was diluted in 90 µl medium. After incubation at 37°C for 1 h, the absorbance was examined at 450 nm using a spectrophotometer (Bio‐Rad Laboratories, Inc.). GraphPad Prism 5.0 program (GraphPad Software, Inc.) was used to construct the cell growth inhibition curve and calculate the IC_50_ value.

### 2.5 Clone survival assay

Cells were transfected and reseeded onto six‐well plates at a density of 800 cells per well for clone survival assay. Next, the cells were treated with different concentrations of irinotecan. Two weeks later, cells were fixed with 4% paraformaldehyde for 20 min and stained with crystal violet for 30 min at room temperature (Beyotime Institution of Biotechnology). The cluster number of each well was counted.

### 2.6 Flow cytometric analysis

In six‐well plates, HT29 and Caco2 cells were seeded and transiently transfected before being exposed to irinotecan for 48 h. The Annexin V‐FITC Apoptosis Detection Kit (Beyotime Institution of Biotechnology) was used to assess cell apoptosis. Cells were first fixed in 70% ethanol at 4°C overnight for cell cycle analysis. The Cell Cycle and Apoptosis Analysis Kit (Beyotime Institution of Biotechnology) was then used to test cell cycle distribution according to the manufacturer's instructions.

### 2.7 Terminal deoxynucleotidyl transferase‐mediated UTP nick‐end labelling (TUNEL) assay

Cell apoptosis was detected using TUNEL staining. In 24‐well plates, HT29 and Caco2 cells were seeded and transiently transfected before being exposed to irinotecan for 48 h. Apoptotic cells were detected with One Step TUNEL Apoptosis Assay Kit (Beyotime). Cells were washed three times with PBS and fixed in 4% paraformaldehyde for 20 min. The cells were then incubated with 0.3% Triton X‐100 for 5 min and stained with TUNEL solution for 1 h at room temperature before being washed gently with PBS. The cell nucleus was labelled by Hoechst staining. The apoptotic cells were observed under a fluorescence microscope.

### 2.8 Alkaline comet assay

Alkaline comet assay was performed using the CometAssay^®^ Kit (Trevigen). Before being subjected to irinotecan, the Caco2 and HT29 cell lines were first transfected. The cells were then suspended in cold PBS and mixed with molten LMAgarose (37°C preheat) at a ratio of 1:10 and quickly pipetted 50 μl was quickly pipetted onto CometSlide™, ensuring that the sample completely covered the sample area. The slides were incubated at 4°C for about 20 min to allow the agarose to gel before being immersed in 4°C Lysis Solution for 1 h. The slides were then transferred into Alkaline Unwinding Solution and incubated at room temperature for 20 min. The slides were subjected to electrophoresed at 21V for 40 min and gently immersed twice in dH_2_O and once in 70% ethanol for 5 min each. Next, the samples were air‐dried and 50 μl diluted SYBR^®^ Gold (Trevigen) was added to every sample and stained in dark place at room temperature for 30 min. The slides were then gently rinsed in water, air‐dried and observed using a fluorescent microscope. CaspLab Software was used to analyse the length of the DNA tails.

### 2.9 Immunofluorescence

HT29 and Caco2 cells were seeded in confocal dish. Cells were fixed in 4% paraformaldehyde for 20 min after transfection and irinotecan treatment and then permeabilized with 0.1% Triton X‐100 at room temperature for 15 min. Then 5% goat serum was used to block non‐specific binding. Cells were incubated at 4°C overnight in primary antibodies diluted in 5% goat serum. Subsequently, cells were placed in the dark and incubated with a fluorescence labelling secondary antibody for 1 h before being stained with DAPI for 15 min. A confocal microscope was used to capture the images.

### 2.10 Co‐Immunoprecipitation (Co‐IP)

Co‐Immunoprecipitation assay was performed using the Pierce Co‐Immunoprecipitation Kit (Thermo Scientific, 26149) following the manufacture's protocol. Briefly, antibodies were immobilized on AminoLink Plus Coupling Resin by rotating at room temperature for 2 h. Cells were lysed on ice using ice‐cold IP Lysis/Wash Buffer and then centrifuged at 13,000 *g* for 10 min. The supernatants were then incubated with bait‐prey protein mixture at 4°C overnight, followed by elution steps. A 5×Lane Marker Sample Buffer was added to the samples buffer, and the samples were heated at 95–100°C for 5 min before being applied to the gel.

### 2.11 Luciferase reporter assay

The eIF3a‐silenced and control HT29 and Caco2 cells were reseeded into 24‐well plate and transfected with RRR2R5A 5’UTR luciferase plasmids along with renilla luciferase vector. After 48 h, the renilla luciferase activity was used as a transfection efficiency control. The Dual Luciferase Reporter Assay Kit (Promega) was used to test the activity of luciferase based on the manufacturer's protocol.

### 2.12 RNA‐binding protein immunoprecipitation (RIP)

The RIP assay was performed using the EZ‐Magna RNA Immunoprecipitation (RIP) Kit (Millipore) following the given instructions. For this experiment, 293T cells were harvest and lysed in ice‐cold lysis buffer containing RNase and protease inhibitor for 20 min. Then the cell lysates were centrifuged and the supernatant was incubated with anti‐rabbit IgG or anti‐rabbit eIF3a antibodies with rotation at 4°C overnight. After that, the immunoprecipitated RNA was isolated and the expression of PPP2R5A mRNA was quantified using RT‐PCR assay.

### 2.13 Animal experiments

For the in vivo irinotecan treatment assay, 40 male BALB/c nude mice (4–5 weeks) were randomly divided into four groups (*n* = 10): control group, eIF3a‐knockdown group, control +irinotecan group and eIF3a‐knockdown +irinotecan group. The shRNA sequence targeting human eIF3a cDNA was purchased from Sigma and listed in Table S3. Stable eIF3a knockdown and control HT29 cell lines were generated. Cells (5 ×  10^6^ per mouse) were resuspended in 100 µl McCoy's 5A medium and subcutaneously injected into the right flanks of nude mice. Mice were observed until the tumour volume reached to approximately 50 mm^3^. Mice in the irinotecan‐treated group were intraperitoneally injected with 5 mg/kg irinotecan twice per week. The length (L) and width (W) of tumour were measured every 3 days. The tumour volumes were calculated as LW^2^/2. Approximately 50 days after injection, mice were euthanized and tumours were embedded in paraffin for further research. The animal studies were approved by the Animal Ethics Committee of the Third Xiangya Hospital of Central South University. All possible methods were conducted to minimize the animal suffering.

### 2.14 Statistical analysis

The statistical analyses were performed using SPSS software (IBM, Inc.) and GraphPad Prism 5 (GraphPad). All assays presented were calculated from three independent experiments. The significant difference between groups was estimated using the student's *t*‐test. A value of *p* <  0.05 was considered statistically significance (*p < 0.05, **p < 0.01, ***p < 0.001, ****p < 0.0001, N.S. no significant difference). All values are presented with mean ± standard deviations (SD).

## 3 RESULTS

### 3.1 eIF3a suppression leads to cellular resistance to irinotecan in colorectal cancer

**FIGURE 1:**
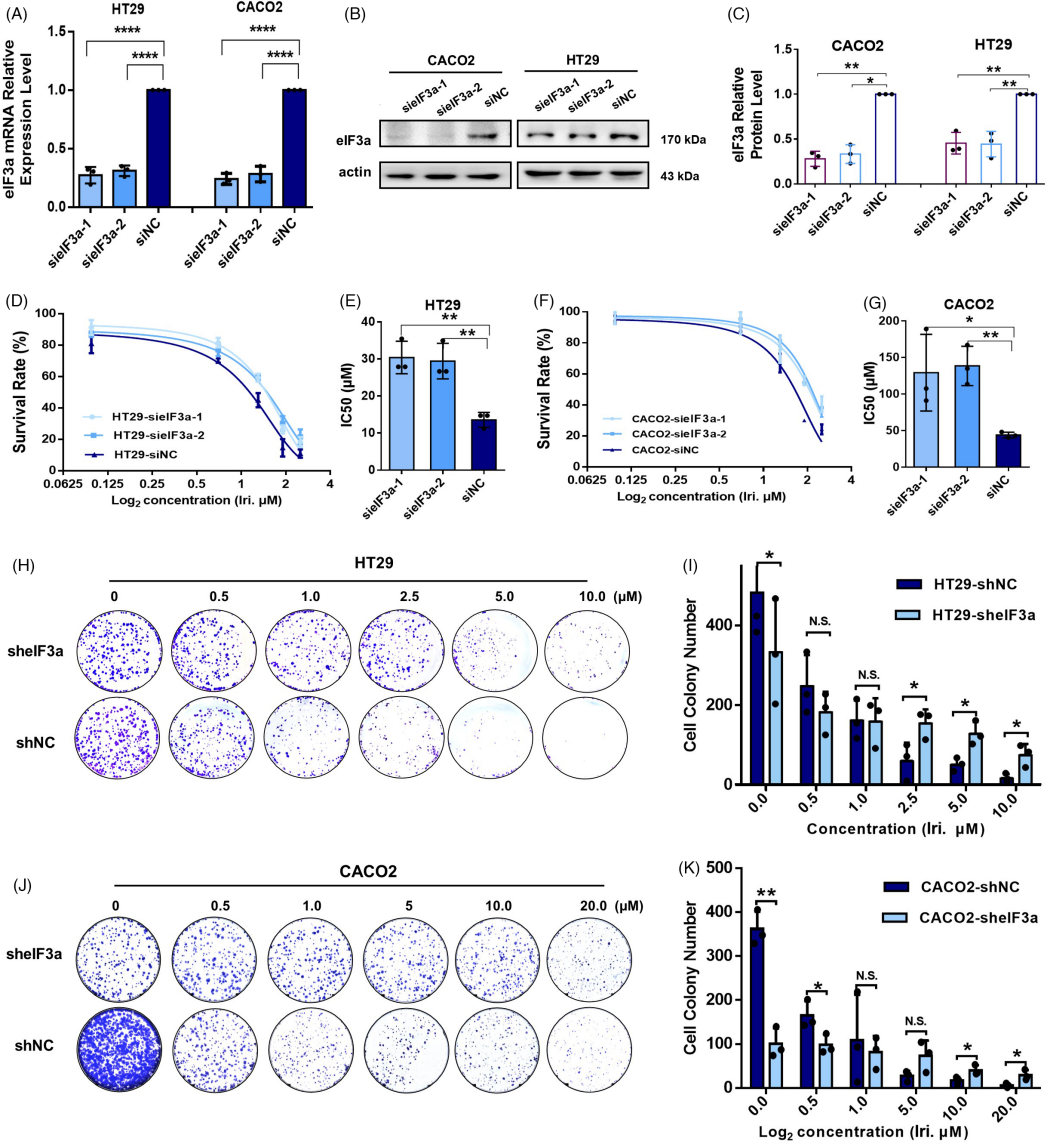
eIF3a suppression induced cellular resistance to irinotecan treatment. (A) eIF3a was downregulated in HT29 and Caco2 cell lines and the knockdown efficiencies of eIF3a mRNA were verified using RT‐PCR assays. The statistical results were calculated from three independent experiments. (B) Western blot assays were carried out to test the knockdown efficiencies of eIF3a protein in HT29 and Caco2 cell lines, respectively. (C) The statistical results of Figure 1B were calculated from three independent experiments. (D–G) eIF3a was downregulated in HT29 and Caco2 cell lines. The CCK8 viability assays were used to measure irinotecan sensitivity changes in response to eIF3a suppression in HT29 (D) and Caco2 (F) cell lines. The IC_50_ values of three independent experiments were statistically analysed and presented. (H–K) Stable eIF3a‐knockdown (sheIF3a) and control (shNC) cell lines were generated and applied to colon formation assay (H, J). The cloned cells were quantified from three independent experiments and statistical analyses were conducted (I, K)

To identify the role of eIF3a in irinotecan sensitivity, the CCK8 cell viability assays were performed in HT29 and Caco2 cells, which were either treated with irinotecan at different doses for 48 h or treated with DMSO as a negative control. The knockdown efficiency of two specific siRNAs (sieIF3a‐1 and sieIF3a‐2) was detected using RT‐PCR and western blot assays in comparison with a negative control (siNC) (Figure 1A–C). It was discovered that eIF3a downregulation significantly increased resistance to irinotecan (Figure 1D,F). The IC_50_ values supported the outcomes of cell viability assays (Figure 1E,G). These results were further confirmed by clone survival assays. The eIF3a stable silenced Caco2 and HT29 cells were generated using short hairpin RNA (shRNA) (Figure 1H–K). It was found that irinotecan treatment significantly inhibited the ability of control cells to form colonies, whereas eIF3a suppression cells showed more resistance.

In consistent with our study, eIF3a knockdown has been reported to inhibit the proliferation of several types of tumour cells.^20^, ^23^ Reduction in cell proliferation may also impact drug resistance. To exclude the possible effect of proliferation inhibition on irinotecan sensitivity, we selected a colorectal cancer cell line, SW620, which showed least sensitive to eIF3a‐knockdown‐induced proliferation inhibition. The amount of siRNA used in transfection process was also cut down to minimize the effect of eIF3a knockdown on SW620 proliferation (Figure S1A,B). As shown in the result, on the premise that eIF3a knockdown had no significant effect on SW620 proliferation (Figure S1C), the impact of eIF3a suppression on irinotecan sensitivity was still significant (Figure S1D). To summarize, the aforementioned results indicated the specific role of eIF3a in irinotecan sensitivity, and provided a potential therapeutic target for irinotecan‐based chemotherapy.

### 3.2 Knockdown of eIF3a increases irinotecan resistance in vivo

**FIGURE 2:**
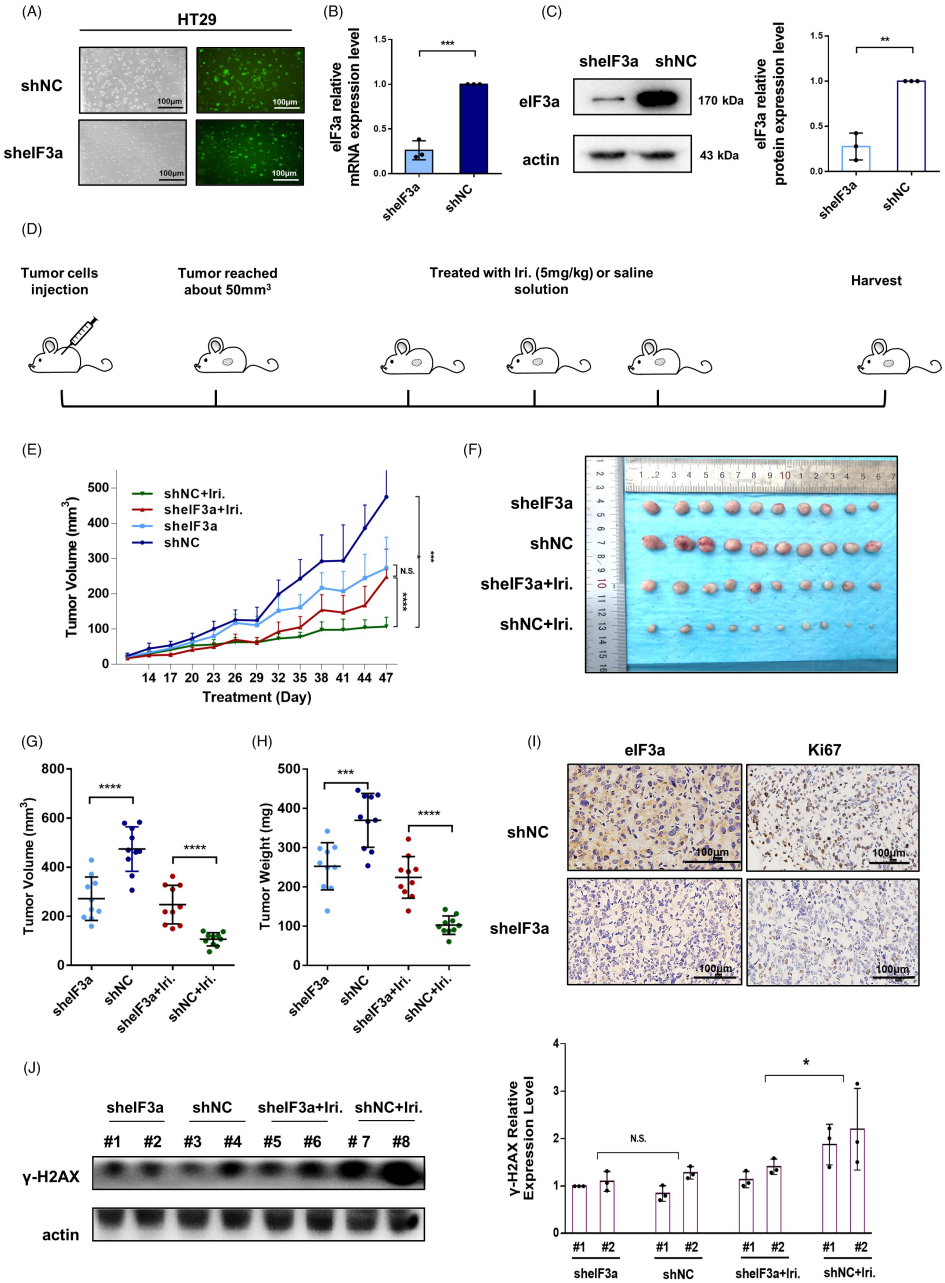
Knockdown of eIF3a increased irinotecan resistance in vivo. (A) Representative images of fluorescence intensity for the generated GFP‐tagged sheIF3a and shNC HT29 cells. (B, C) The q‐PCR and western blot assays were performed for the verification of eIF3a‐knockdown efficiency in mRNA (B) and protein level (C), respectively. (D) A flow chart of in vivo irinotecan sensitivity assay. (E) A line chart exhibiting the tumour volumes of each mouse measured every 3 days. The results were shown as mean ± SD. The statistical analysis reflected the difference of tumour volumes at the end point. (F) The mice were euthanized after about 50 days of injection. Tumours of each mouse were separated and presented. (G,H) The tumour volumes (G) and weights (H) were measured and exhibited as mean ± SD. (I) Representative images of immunohistochemical staining assay of tumours. (J) The protein samples of tumour tissues were extracted from mouse tumours. Two tumour protein samples were collected from each group for western blot analysis. The expression of γ‐H2AX were tested. The statistical results were calculated from three independent experiments. Iri: irinotecan

To study the role of eIF3a in irinotecan sensitivity in vivo, HT29 cells were engineered with eIF3a stable knockdown (sheIF3a) and negative control (shNC) to construct nude mouse xenograft model (Figure 2A–C). When the tumour volume reached about 50 mm^3^, tumor‐bearing mice in the experimental and control groups were intraperitoneally treated with irinotecan or saline (contained DMSO) twice a week (Figure 2D). The tumour volumes were measured every 3 days (Figure 2E). All mice were euthanized at 50 days after cell injection and tumours were separated for further investigation (Figure 2F). Tumor volumes and weights were measured (Figure 2G,H). As shown in the results, the tumour growth rate in the control group was faster than in the eIF3a‐silencing group, demonstrating that eIF3a promotes proliferation, which was consistent with previous findings.^20^, ^23^ However, when treated with irinotecan, tumours in eIF3a‐silencing group exhibited significant drug resistance. These outcomes were consistent with those of our in vitro experiments. The IHC staining assays on eIF3a and Ki67 revealed the successful knockdown of eIF3a (Figure 2I). The downregulation of Ki67 also partly reflected the successful knock down of eIF3a. Then the proteins were extracted from mouse tumours and the γ‐H2AX expression was tested by western blot assay. As shown in Figure 2J, the γ‐H2AX expression was significant higher in tumours in shNC+Iri. group than sheIF3a+Iri group, suggesting a more serious DNA damage degree. These results indicated that eIF3a downregulation accelerates cellular resistance to irinotecan in vivo, affirming the essential role of eIF3a in irinotecan sensitivity.

### 3.3 eIF3a silencing reduces irinotecan‐induced cell apoptosis

**FIGURE 3:**
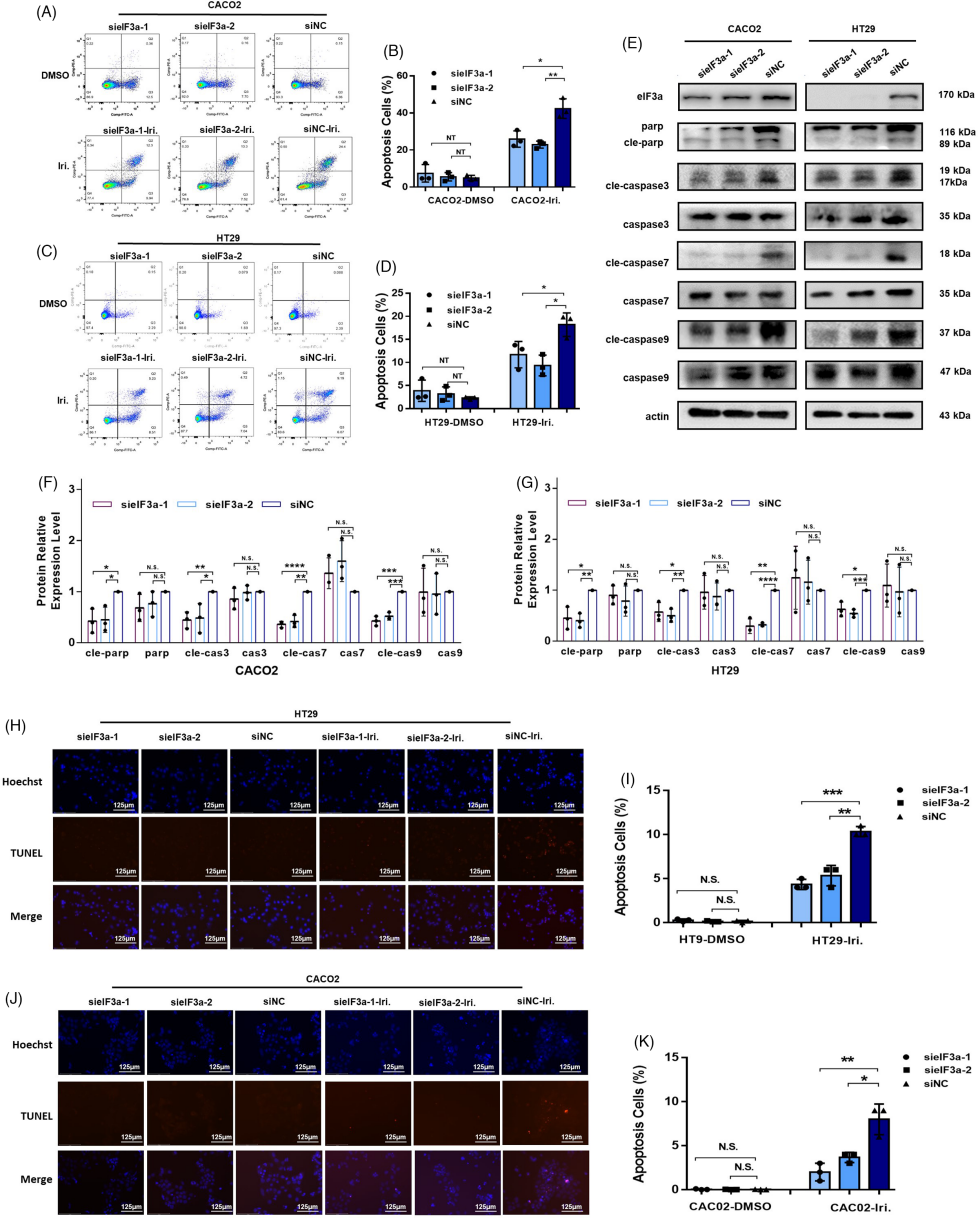
eIF3a suppression significantly reduced irinotecan‐induced cell apoptosis. (A–D) The eIF3a silencing and control Caco2 (A) and HT29 (C) cells were exposed to irinotecan for 48 h and then subjected to flow cytometry assay. Three independent experiments were conducted, and statistical results were presented (B, D). (E) The eIF3a silencing and control Caco2 and HT29 cells were exposed to irinotecan for 48 h and proteins were extracted for western blot assay. Key proteins in the apoptosis pathway were detected. (F,G) The statistical results of the western blot assay shown in Figure 3E, which were calculated from three independent experiments. (H–K) eIF3a was silenced in HT29 and CACO2 cells. Then cells were exposed to irinotecan for 48 h and TUNEL assays were conducted to detect irinotecan‐induced apoptosis in HT29 (H, I) and Caco2 (J, K) cells. Hoechst staining was used to label cell nucleus. Representative images (H, J) were exhibited. Figure I, K showed the percentage of positive cells statistically analysed from three independent experiments. Iri: irinotecan

Subsequently, we investigated irinotecan‐induced tumour cell apoptosis in the presence of eIF3a silencing or not. Flow cytometry assays were performed to detect apoptotic cells. The results showed that eIF3a knockdown had no obvious impacts on malignant cell apoptosis but significantly decreased irinotecan‐induced apoptosis in both cell lines (Figure 3A–D). We also evaluated the changes in key proteins that participated in apoptotic signalling (Figure 3E). There was obvious decrease in protein expression of cleaved parp, cleaved caspase‐3, cleaved caspase‐7 and cleaved caspase‐9 in eIF3a‐silencing cells (Figure 3F,G). Furthermore, the TUNEL staining assays consistently demonstrated that eIF3a suppression lowered the apoptosis rate (Figure 3H–K). To summarize, eIF3a significantly affects irinotecan‐induced colorectal cancer cell apoptosis.

### 3.4 eIF3a affects irinotecan‐induced DNA damage and γ‐H2AX foci formation

**FIGURE 4:**
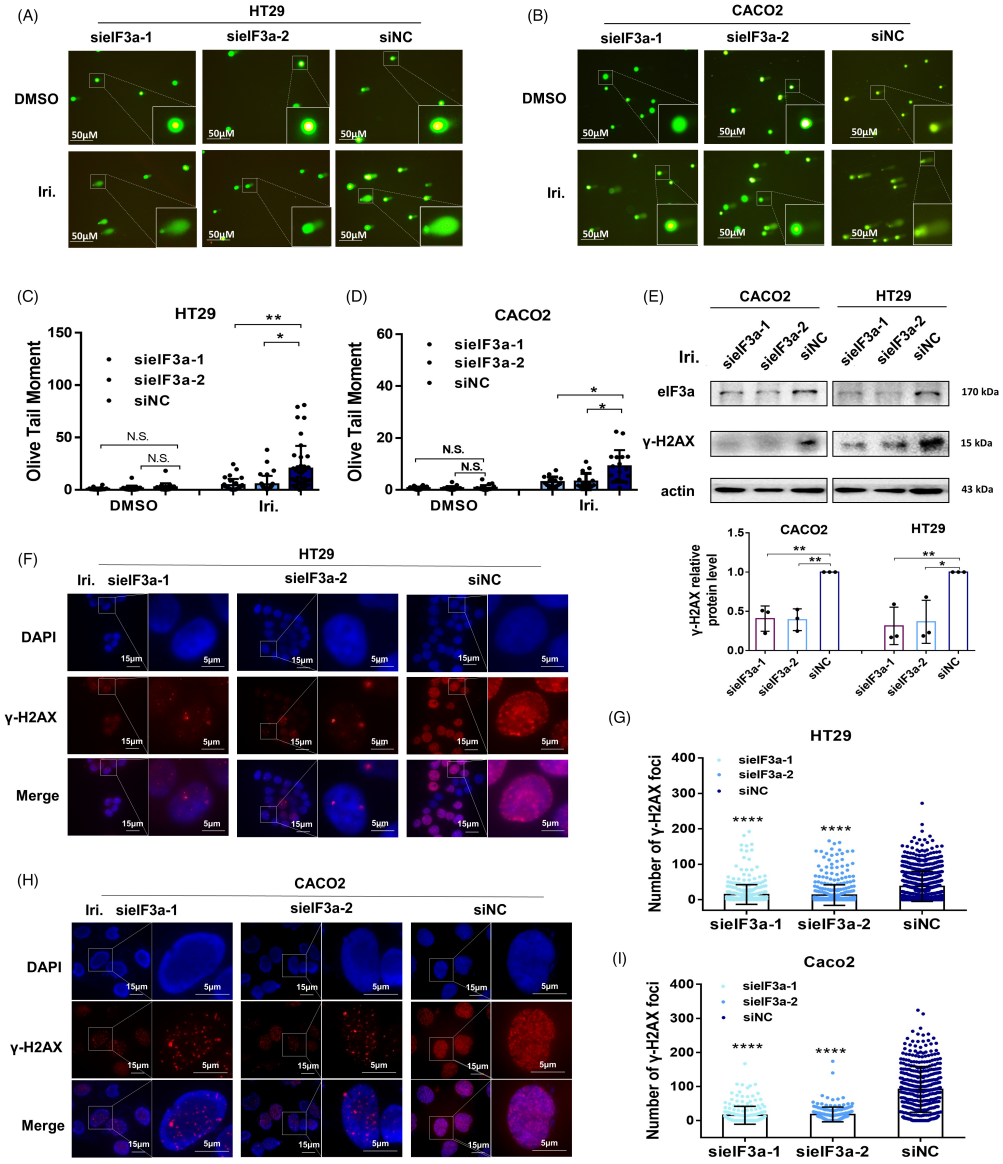
eIF3a silencing decreased irinotecan‐induced DNA strand breaks and γ‐H2AX formation. (A–D) Irinotecan was administered to HT29 and CACO2 cells that had been transfected with eIF3a or control siRNAs. The alkaline comet assay was used to detect the DNA SSBs formation in HT29 (A, C) and Caco2 (B, D) cells. The length of comet tails was measured and presented as mean ± SD. (E) The eIF3a‐silencing and control Caco2 and HT29 cells that were exposed to irinotecan for 48 h. The protein expression level of γ‐H2AX was tested by western blot assay. (F–I) Immunofluorescence assays were performed in HT29 (F, G) and Caco2 (H, I) cells. The eIF3a expression was silenced in HT29 and Caco2 cells and then cells were treated with irinotecan for 48 h. The numbers of γ‐H2AX positive foci in each cell unclears were counted using Image J software. More than 200 unclears were counted in each group. The result was presented as mean ± SD (G, I)

It is generally agreed that the major anti‐tumor mechanism of irinotecan is to generate DNA strand breaks. To visualize the irinotecan‐induced DNA damage, we performed alkaline comet assay to identify SSBs formed in the nucleus of tumour cells. The length of comet tails shows the severity of DNA damage, and we observed a significant reduction in DNA damage in eIF3a‐silenced cells after 48 h of irinotecan treatment (Figure 4A–D). When the DNA SSBs encounter the replication forks, they would convert to DSBs. Therefore, western blot assays were used to test γ‐H2AX, a widely recognized indicator of DSBs (Figure 4E). It was discovered that irinotecan‐induced γ‐H2AX formation was notably attenuated in eIF3a‐knockdown cells. Moreover, the immunofluorescence assays were carried out to visualize the γ‐H2AX foci formation in a more intuitive way. We quantified the γ‐H2AX focus in each cell nucleus and found a significant reduction in γ‐H2AX formation in eIF3a‐knockdown cells (Figure 4F–I). In consistent with previous experiments, these results indicated that eIF3a silencing reduces nuclear DNA damage after irinotecan exposure.

### 3.5 eIF3a regulates irinotecan‐induced G2/M cell cycle arrest

**FIGURE 5:**
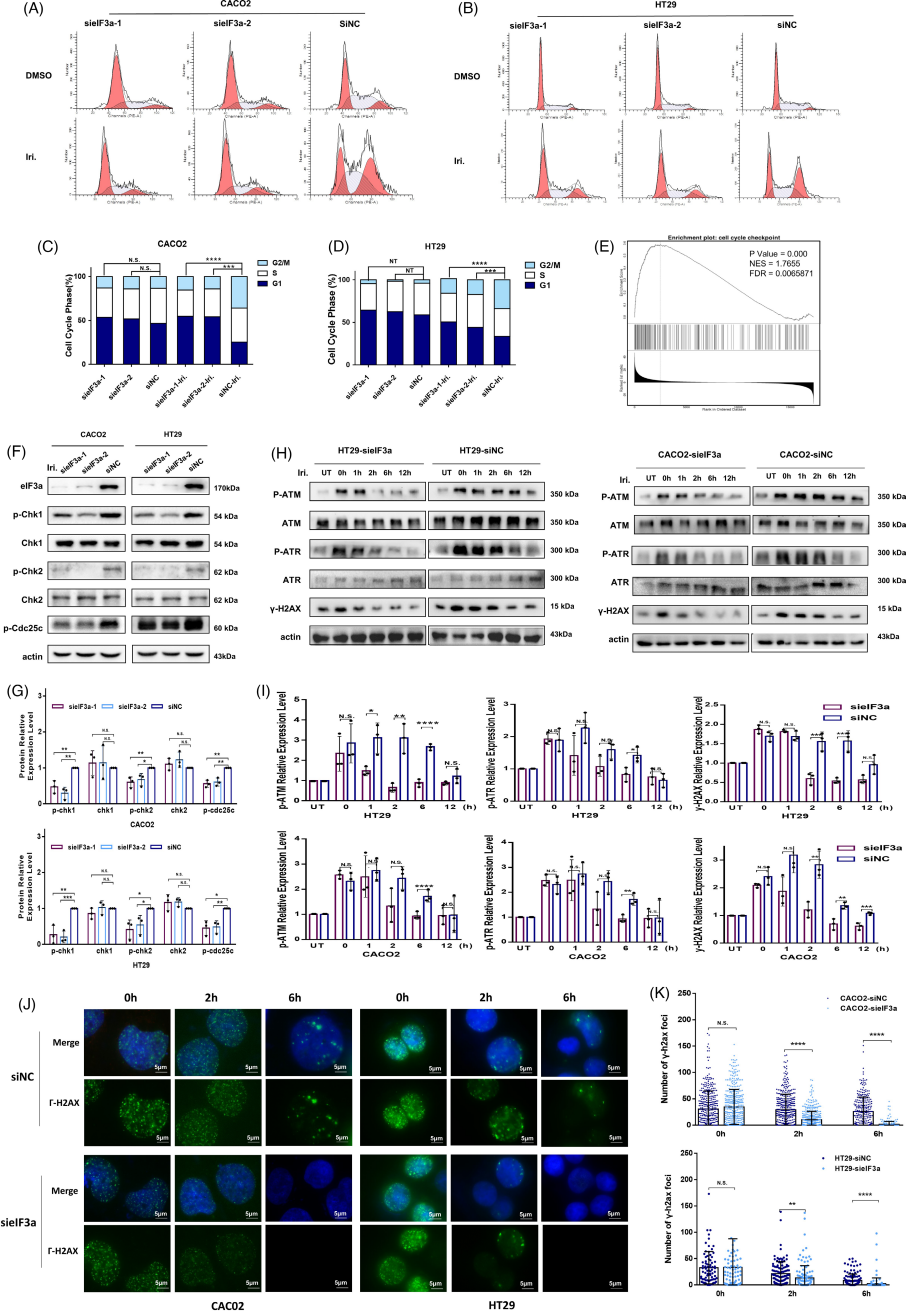
eIF3a silencing reduced G2/M cell cycle arrest and persistent ATM/ATR phosphorylation. (A–D) Irinotecan was administered to eIF3a‐knockdown and control Caco2 (A, C) and HT29 (B, D) cells for 48 h before being subjected to flow cytometry assays. Three independent experiments were performed and the cell cycle distribution was analysed (C, D). (E) GSEA analysis was performed online at the LinckedOmics database (http://www.linkedomics.org/) by taking use of colorectal cancer dataset. (F, G) The eIF3a‐silenced and control Caco2 and HT29 cells were exposed to irinotecan. The phosphorylation of Chk1, Chk2 and Cdc25c was determined using western blot assay. (H, I) The eIF3a‐silenced and control Caco2 and HT29 cells were exposed to irinotecan for 1 h and then left to recover for the indicated time. The expression of γ‐H2AX, p‐ATM (S1981) and p‐ATR (T1989) at different recovery period were monitored using western blot assay (H). The statistical results of p‐ATM, p‐ATR and γ‐H2AX were quantified from three independent experiments. The relative expression of p‐ATM and p‐ATR expression were normalized to the respective total protein expression (I). (J) eIF3a‐silenced and control Caco2 and HT29 cells were exposed to irinotecan for 1 h and then left to recover for 0 h, 2 h and 6 h. The immunofluorescence assay was used to visualize the γ‐H2AX foci formation. (K) The number of γ‐H2AX positive foci in each cell unclears were counted using Image J software. The result was presented as mean ± SD

Camptothecin and its derivatives have been reported to induce irreversible cell cycle arrest at G2/M phase by regulating the phosphorylation of checkpoint kinase 1 (Chk1) and checkpoint kinase 2 (Chk2).^24^ Flow cytometry assays were used to analyse the cell cycle distribution in colorectal cancer cell lines. HT29 and Caco2 cells were transfected with eIF3a or control siRNAs and exposed to 5 μM irinotecan for 48 h. Irinotecan was found to induce more pronounced accumulations of cells in G2/M phase in the siNC group than in the eIF3a suppression group (Figure 5A–D). Gene set enrichment analysis (GSEA) performed at the LinckedOmics database (http://www.linkedomics.org/) revealed that the cell cycle checkpoint signal pathway was significantly enriched in colorectal cancer patients with higher expression of eIF3a (Figure 5E). To verify these results, a western blot assay was used to examine the impact of eIF3a suppression on key regulatory proteins involved in G2/M cell cycle arrest (Figure 5F,G). In consistent with previous outcomes, the phosphorylation levels of Chk1 and Chk2 were significantly decreased after eIF3a suppression, indicating a mild cell cycle arrest.

### 3.6 eIF3a silencing reduces persistent ATM and ATR phosphorylation

In the light of our findings that eIF3a induced an increase in γ‐H2AX and DNA lesions, we considered the possibility that eIF3a also contributes to DNA damage response. The activation of ATM and ATR is an early event in response to DNA lesions, and both are necessary for initiating DNA damage response cascade, which includes cell cycle arrest and γ‐H2AX signalling. The characters of ATM and ATR upon DNA damage were consisted of a cascade of reactions, including activation by autophosphorylation and progressive deactivation after DNA repair completion. Following the repair process, it was necessary to eliminate the phosphorylated ATM and ATR to complete the repair process, which was followed by the release of cells from growth arrest. Dephosphorylation defect causes persistent activation of ATM/ATR signal and disordered DNA damage repair.^25^, ^26^

To investigate the exact role of eIF3a in ATM and ATR signal, we performed western blot and immunofluorescence experiments to monitor the dynamic process of ATM and ATR signal. Both eIF3a silencing and control HT29 and Caco2 cells were exposed to irinotecan for 1 h before the drug was withdrawn and cells were allowed to recover for the indicated time. The expression of p‐ATM, P‐ATR and γ‐H2AX was tested to address the progress of the repair process in the presence of eIF3a suppression or not. As shown in Figure 5H,I, the ATM/ATR signal was rapidly activated in both cells in response to irinotecan. However, the dephosphorylation of p‐ATM and p‐ATR occurred earlier in eIF3a suppression cells. The γ‐H2AX foci formation were also visualized using immunofluorescence. The γ‐H2AX foci were activated in both eIF3a‐silencing and control cells after irinotecan treatment. With the extension of time, γ‐H2AX foci disappeared more rapidly in eIF3a‐silencng cells (Figure 5J,K). Taken together, these results confirmed that eIF3a participates in the deactivation step of ATM/ATR signal, which is also an indispensable step in an effective and complete DNA damage response.

### 3.7 eIF3a negatively regulates PPP2R5A at transitional level

**FIGURE 6:**
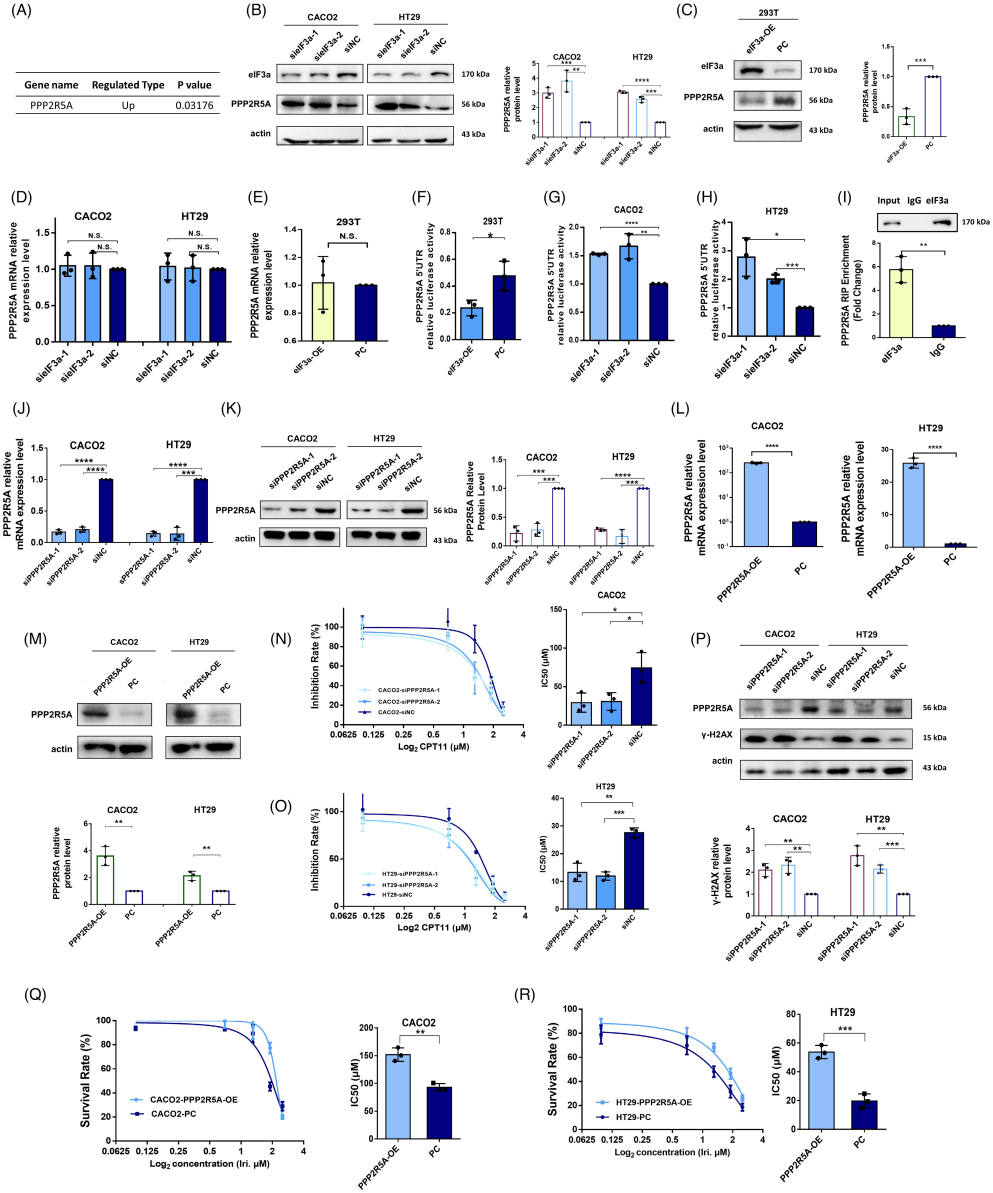
eIF3a translationally regulated PPP2R5A. (A) The mass spectrometry results of the differential expression of PPP2R5A in eIF3a‐silencing and control cells. (B) eIF3a was knocked down in the Caco2 and HT29 cell lines. The protein expression level of PPP2R5A was tested by western blot assay. (C) eIF3a was overexpressed in 293T cell line and protein expression level of PPP2R5A was detected by western blot assay. (D) eIF3a was knocked down in Caco2 and HT29 cell line and the mRNA expression of PPP2R5A was tested by q‐PCR assay. (E) eIF3a was overexpressed in the 293T cell line and the mRNA expression of PPP2R5A was tested by q‐PCR assay. F‐H. eIF3a was overexpressed in 293T cells (F) or silenced in HT29 and CACO2 cells (G,H). The translational activation ability of eIF3a on the 5’UTR region of PPP2R5A was determined using luciferase reporter gene assays. (I) RIP analysis was performed on 293T cells. The direct binding of eIF3a protein to the PPP2R5A mRNA was confirmed. (J) PPP2R5A was silenced in CACO2 and HT29 cells, respectively. The suppression efficiency of PPP2R5A mRNA was confirmed using RT‐PCR assay. (K) The suppression efficiency of PPP2R5A protein was confirmed using a western blot assay. (L,M) PPP2R5A was overexpressed in HT29 and CACO2 cells and the overexpress efficiency was tested by q‐PCR (L) and western blot (M) assays. (N,O) PPP2R5A was silenced in Caco2 and HT29 cell lines before the cell lines were exposed to different doses of irinotecan for 48 h. Cellular sensitivity of irinotecan in response to PPP2R5A suppression was assessed using a CCK8 viability assay. The IC_50_ values were calculated from three independent experiments. (P) The expression of γ‐H2AX was tested in PPP2R5A‐silenced and control Caco2 and HT29 cells. (Q,R) PPP2R5A was overexpressed in Caco2 and HT29 cell lines before the cell lines were exposed to different doses of irinotecan for 48 h. Cellular sensitivity of irinotecan in response to PPP2R5A overexpression was assessed using a CCK8 viability assay. The statically analysis of IC_50_ values were derived from three independent experiments. PC: control plasmid

It has been reported that ATM and ATR could be directly dephosphorylated by a series of phosphatase. For example, PPP2R2A, one of the regulatory subunits of the protein phosphatase 2A (PP2A), was reported to affect Chk2 activity by regulating ATM dephosphorylation at S367, S1893 and S1981.^27^, ^28^ To reveal the putative regulatory mechanism of eIF3a on ATM and ATR phosphorylation, we sought to identify the phosphatase that may participate in this process. Mass spectrometry outcomes of eIF3a‐silencing cells revealed that PPP2R5A is significantly upregulated in response to eIF3a suppression (Figure 6A). This raised the possibility that PPP2R5A may play a key role in eIF3a‐mediated ATM/ATR signalling.

To examine this possibility, we first verified whether eIF3a affected PPP2R5A expression. We performed western blot assay to test PPP2R5A expression in control and eIF3a‐knockdown colorectal cancer cells. Figure 6B shows that PPP2R5A was notably upregulated in response to eIF3a suppression. Next, we overexpressed eIF3a in the 293T cell line and PPP2R5A expression decreased as expected (Figure 6C). PPP2R5A mRNA expression was also evaluated. However, regardless of whether eIF3a was up‐ or down‐regulated, there were no significant differences in PPP2R5A mRNA expression levels (Figure 6D,E). Based on this observation and previous studies that suggested that eIF3a is widely regarded as a translation initiation factor, we hypothesized that the regulatory effect occurs during the translational process. To test this hypothesis, the eIF3a expression was suppressed in HT29 and CACO2 cells, and upregulated in 293T cells, respectively, and the luciferase reporter gene assays were used to examine the translational activation activity of eIF3a on PPP2R5A. The results indicated that in response to eIF3a suppression or overexpression, the luciferase activities of 5′UTR region of PPP2R5A were significantly increased in Caco2, HT29 cells (Figure 6F,G) and decreased in 293T cells (Figure 6H), respectively. Finally, the RIP assay confirmed the direct interaction between eIF3a protein and PPP2R5A mRNA (Figure 6I). Taken together, we proved that eIF3a translationally inhibits PPP2R5A expression, which may be a critical step in the regulatory role of eIF3a in ATM/ATR signalling.

### 3.8 Silencing of PPP2R5A increases cellular sensitivity to irinotecan

To add further supports to the finding that PPP2R5A is needed for eIF3a‐mediated DNA damage response, we investigated whether PPP2R5A affects irinotecan sensitivity. We subsequently knocked down or overexpressed PPP2R5A in the HT29 and Caco2 cell lines. The transfection efficiency was verified at both mRNA and protein levels (Figure 6J–M). The CCK8 cell viability assays were used to test the cellular response to irinotecan, and the IC_50_ values were calculated. In contrast to eIF3a deficiency, silencing PPP2R5A significantly increased cellular sensitivity to irinotecan (Figure 6M–O). The γ‐H2AX formation was also evaluated and an obvious upregulation of γ‐H2AX was found in PPP2R5A‐silenced cells compared to control cells (Figure 6P). In contrast, when the PPP2R5A was overexpressed, HT29 and CACO2 cells became more resistant to irinotecan treatment (Figure 6Q,R). In conclusion, these findings suggested that PPP2R5A directly regulates cellular response to irinotecan treatment.

### 3.9 PPP2R5A suppression leads to prolongs ATM/ATR signal activation

**FIGURE 7:**
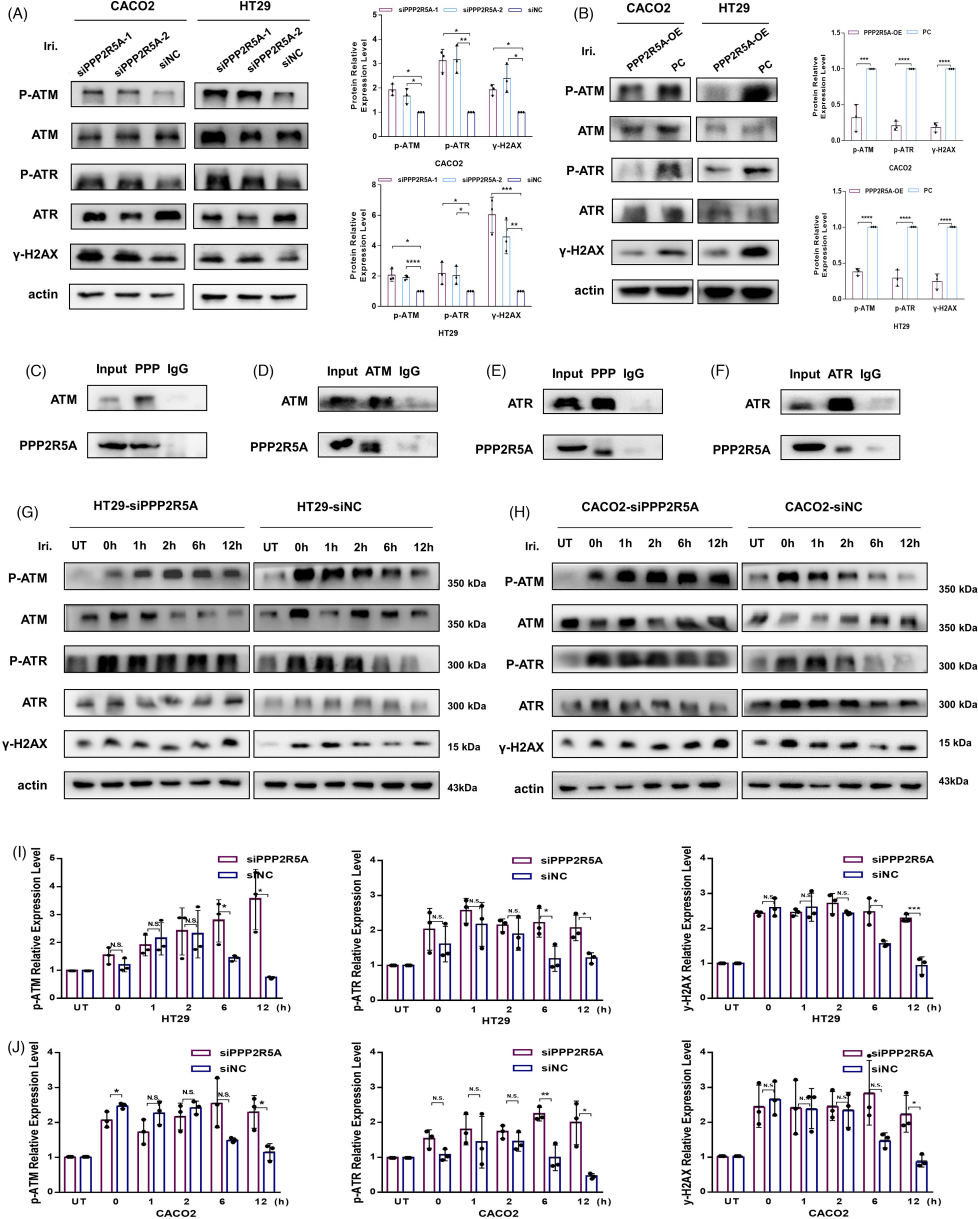
PPP2R5A directly regulates the dephosphorylation of ATM and ATR. (A) PPP2R5A‐silenced and control Caco2 and HT29 cells were treated with irinotecan for 48 h and then examined using western blot assays. The expression of p‐ATM (S1981) and p‐ATR (T1989) was tested. The relative expression of p‐ATM and p‐ATR expression were normalized to the respective total protein expression. (B) PPP2R5A‐overexpressed and control Caco2 and HT29 cells were treated with irinotecan for 48 h and the expression of p‐ATM (S1981) and p‐ATR (T1989) was tested by western blot assays. The relative expression of p‐ATM and p‐ATR expression were normalized to the respective total protein expression. (C–F) The co‐IP assays were used to verify the interaction between PPP2R5A with ATM (C, D) and ATR (E, F). G‐J. PPP2R5A‐silenced and control Caco2 and HT29 cells were exposed to irinotecan for 1 h allowed to recover for the indicated time. The expression of γ‐H2AX, p‐ATM and p‐ATR at different recovery times was monitored using a western blotting assay (G, H). The relative expression of p‐ATM and p‐ATR expression were normalized to the respective total protein expression. The statistical results were derived from three independent experiments (I, J)

We then studied whether PPP2R5A plays a role in ATM and ATR signalling. Western blot analyses were used to test the phosphorylation statues of ATM and ATR in cells treated with irinotecan for 48 h. As shown in Figure 7A, a significant upregulation of phosphorylated ATM/ATR and γ‐H2AX was observed after PPP2R5A silencing, while contrary results were found in PPP2R5A overexpression cells (Figure 7B). We also performed a co‐immunoprecipitation assay, which verified the positive interactions between endogenous PPP2R5A with ATM and ATR (Figure 7C–F). These observations raised the possibility that PPP2R5A regulates the ATM/ATR signal.

To investigate the exact regulatory role of PPP2R5A and the overall process of irinotecan‐activated ATM/ATR signalling, including phosphorylation and dephosphorylation, we exposed colorectal cancer cells to irinotecan for 1 h and then left the cells to recover for different time (Figure 7G–J). According to the control cells, p‐ATM and p‐ATR were all rapidly activated and phosphorylated in response to irinotecan treatment. Then, as time passed and repair process progressed, p‐ATM and p‐ATR were gradually dephosphorylated, and the cells resumed normal growth cycle. However, in PPP2R5A‐silenced cells, p‐ATM, p‐ATR and γ‐H2AX remained phosphorylated, indicating a prolonged DNA damage response and impaired DNA repair process. To summarize, these results demonstrated that PPP2R5A participates in irinotecan‐induced DNA damage response by directly regulating the dephosphorylation of p‐ATM and p‐ATR.

## 4 DISCUSSION

The continuously increasing incidence and motility of colorectal cancer pose a severe threat to human health.^29^, ^30^ Recent advances in individualized medicine and pharmacogenomics have achieved certain success. However, the 5‐year survival rate of patients with advanced disease remains poor.^31^ Therefore, the discovery of effective biomarkers and the reinforcement of precision pharmaceutical care based on individual differences are the current research priorities. In this study, we showed that eIF3a may be responsible for cellular sensitivity of irinotecan in vivo and in vitro. High eIF3a expression correlates with better therapy outcomes. It will be interesting to verify this correlation in a clinical study, as it could provide a novel potential biomarker for identifying susceptible individuals who could benefit from irinotecan therapy.

When exposed to DNA‐damaging agents, mammalian cells trigger defensive signals by activating a series of proteins involved in cell cycler checkpoint, DNA damage repair, protein recruitment and degradation. An aberrant repair ability enables malignant cells to survive from DNA‐damaging agents, which can result in serious drug resistance and tumour development. Both ATM and ATR are pivotal kinases in DNA damage response, which integrate DNA damage signals and the cellular responsive mechanism via phosphorylation of multiple downstream factors.^32^ The autophosphorylation of ATM and ATR in response to DNA lesions is an early event that induces and activates a series of downstream signals.

To date, the activation of ATM/ATR signal has been intensively studied. However, how ATM and ATR are dephosphorylated and eliminated and the consequence of persistent activation of p‐ATM and ATR remains largely unknown. Long‐term phosphorylation of ATM and ATR causes aberrant activation of downstream effectors, and, finally, the cell death through apoptosis,^33^ indicating the indispensable benefit of timely removal and deactivation of ATM and ATR in effective DNA repair and cell survival. In this study, we demonstrated for the first time that eIF3a expression is positively correlated with better irinotecan chemotherapy sensitivity both in vivo and in vitro. In‐depth mechanism studies revealed that eIF3a silencing induced significant alterations in the G2/M cell cycle checkpoint, cell apoptosis and DNA damage degree due to changes in ATM and ATR phosphorylation levels.

Therefore, identifying the critical phosphatase involved in eIF3a‐regulated ATM and ATR phosphorylation became the next research priority. After screening all phosphatases identified in our previous mass spectrometry assay and searching and reviewing related literatures, we focused on PPP2R5A, a substrate of PP2A. As a highly conserved eukaryotic phosphatase of the PPP family, PP2A accounts for the majority of Ser/Thr phosphatase activities.^34^ PPP2R5A, one of the regulatory subunits of the PP2A complex, has been linked to a variety of cellular activities.^35^ Abnormality of PPP2R5A is often associated with many diseases, including several types of tumours.^36^ PPP2R5A has been reported to interact with CDK and CHK, which are the necessary proteins involved in checkpoint response induced by DNA damages.^37^, ^38^ PPP2R5A also regulates the degradation of γ‐H2AX in order to avoid prolonged γ‐H2AX‐induced hypersensitive, persistent, but inefficient DNA repair.^39^ All the aforementioned researches demonstrated the special role of PPP2R5A in cell survival and DNA damage response, and raised the possibility that PPP2R5A may play a key role in eIF3a‐mediated ATM/ATR signalling.

Thus, we tested our hypothesis that PPP2R5A participates in the eIF3a‐regulated ATM and ATR signalling. Consequently, we proved that PPP2R5A directly dephosphorylated and deactivated p‐ATM and p‐ATR, which contributed to the timely removal and deactivation of ATM/ATR signal after the repair process was completed.

In conclusion, this study identified a previously unknown mechanism by which eIF3a regulates irinotecan sensitivity. In response to irinotecan‐induced DNA lesions, eIF3a participates in the deactivation of ATM/ATR signal by translationally regulating PPP2R5A expression. We also demonstrated that PPP2R5A can directly interact with and facilitate the dephosphorylation of p‐ATM and p‐ATR after the DNA repair process is completed. Suppression of PPP2R5A prolongs ATM/ATR signal activation and impairs the DNA repair process. Our study may contribute substantially to the early identification of patients who may benefit from irinotecan‐based chemotherapy and the provision of personalized medication to achieve better outcomes. We also provided a new perspective into the dynamic regulation of the ATM/ATR signal, which offered new evidence to target eIF3a as an essential factor in the irinotecan‐induced DNA damage response. In line with our previous investigations, eIF3a correlated with the prognosis and outcome of clinical cancer therapy. Further clinical research is now imperative, and it may eventually aid in the discovery of new potential cancer therapeutic drugs.

## CONFLICT OF INTEREST

The authors declare no conflict of interest.

## AUTHOR CONTRIBUTIONS

CM conceived the study, performed experiments, analysed data and wrote the manuscript. ZS performed experiments and analysed the data. LT, JG and XL reviewed the manuscript. ZL conceived the study and reviewed the manuscript.

## Supporting information

Fig S1

Table S1‐3

## Data Availability

All data used in this study are included in this manuscript and the supplementary information files.
