# Supplementary figures and images for: eIF3a‐PPP2R5A‐mediated ATM/ATR dephosphorylation is essential for irinotecan‐induced DNA damage response
Source: Cell Prolif. 2022 Feb 21;55(4):e13208. doi: 10.1111/cpr.13208 (PMC9055905; doi:10.1111/cpr.13208)

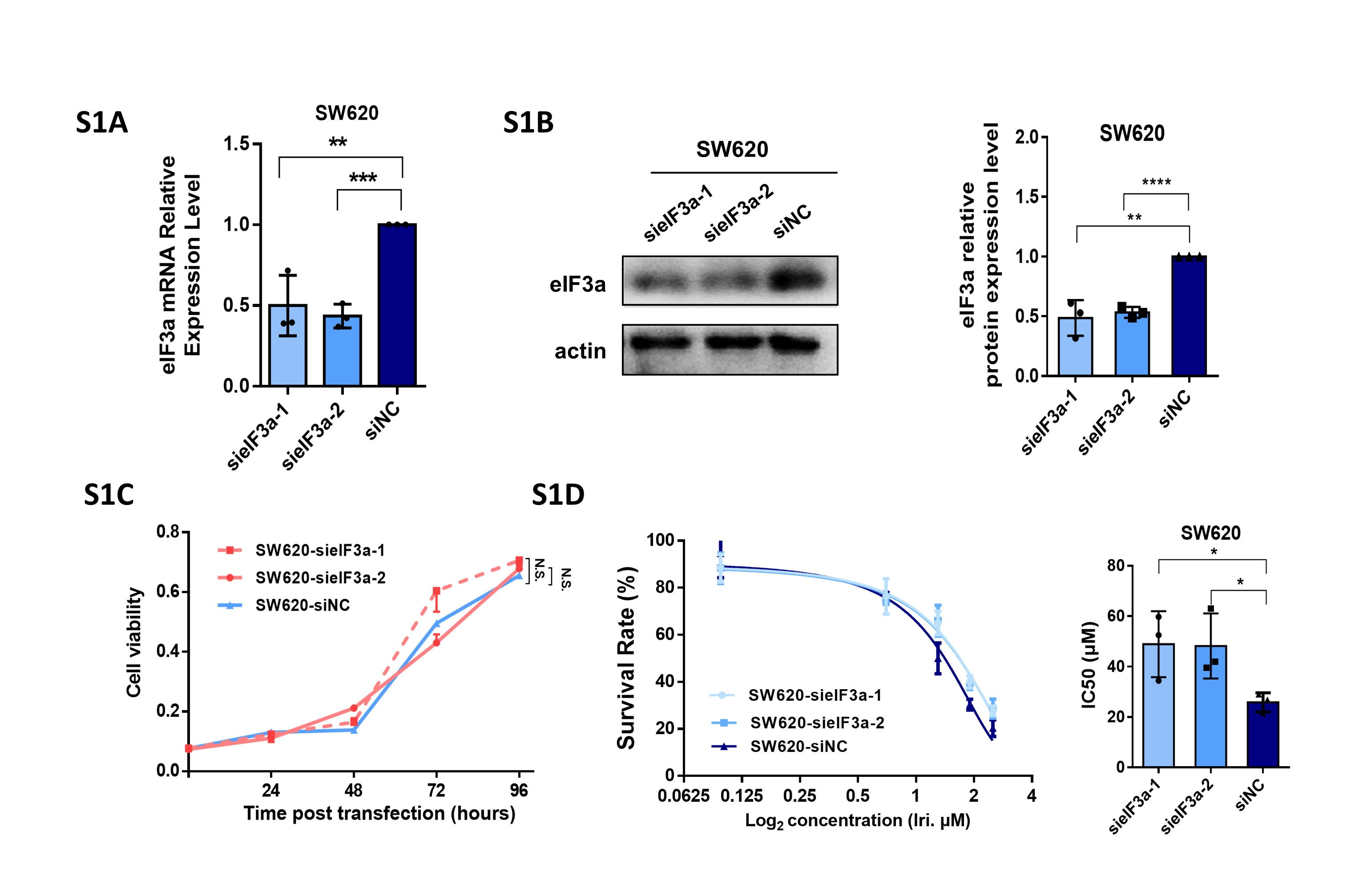

Supplement: Supplementary file 1 — Fig S1 [file CPR-55-e13208-s002.jpg]
