# Supplementary material for: eIF3a‐PPP2R5A‐mediated ATM/ATR dephosphorylation is essential for irinotecan‐induced DNA damage response
Source: Cell Prolif. 2022 Feb 21;55(4):e13208. doi: 10.1111/cpr.13208 (PMC9055905; doi:10.1111/cpr.13208)
Supplement: Supplementary file 2 — Table S1‐3 [file CPR-55-e13208-s001.docx]

| **Supplementary Table 1. siRNA sequences of candidate genes for knockdown** | |
| --- | --- |
| **Gene** | **Sequences** |
| eIF3a | si1: 5'-CGTGCTGATGATGATCGGTTT-3' |
|  | si2: 5'-GCGCCTTGAGAGTCTGAATAT-3' |
| PPP2R5A | si1: 5'-GGTATACAATGTGCTGAAA-3' |
|  | si2: 5'-GAATCAGCGTATTCTGATA-3' |

| **Supplementary Table 2. Primers of candidate genes for RT-PCR** | |
| --- | --- |
| **Gene** | **Sequences** |
| eIF3a | F: 5'-TCAAGTCGCCGGGACGATA -3' |
|  | R: 5'-CCTGTCATCAGCACGTCTCCA-3' |
| PPP2R5A | F:5'-AGAGCCCTGATTTCCAGCCTA-3' |
|  | R:5'-TTTCCCATAAATTCGGTGCAGA-3' |
| beta-actin | F: 5'-TGACTGACTACCTCATGAAGAT-3' |
|  | R: 5'-CATGATGGAGTTGAAGGTAGTT-3' |

| **Supplementary Table 3. shRNA sequences of candidate genes for knockdown** | |
| --- | --- |
| **Gene** | **Sequences** |
| eIF3a | sh1: CGTGCTGATGATGATCGGTTT |
